# Nursing students’ attitudes and beliefs regarding sexual healthcare in Ethiopia: An online cross-sectional study

**DOI:** 10.1371/journal.pone.0278760

**Published:** 2022-12-07

**Authors:** Kalid Seid, Tesfaye Kebede, Nigatu Dessalegn, Yetemegnehuat Ejara, Fikre Moga, Matusala Daniel, Nuredin Mohammed, Seid Hassen

**Affiliations:** 1 Department of Nursing, College of Medicine and Health Sciences, Mizan-Tepi University, Mizan, Southwest People Regional State, Ethiopia; 2 Department of Nursing, College of Medicine and Health Sciences, Arba Minch University, Arba Minch, Ethiopia; Bilawal Medical College, Liaquat University of Medical and Health Sciences, PAKISTAN

## Abstract

**Background:**

Unresolved sexual issues can have an impact on a person’s wellbeing, social interactions, or even medication compliance. Given the enormous global demand to enhance and preserve sexual health, faculty members have a specific goal of developing nursing workforce who are truly prepared to work with clients who have sexual health issues. Hence, the study’s purpose has been to evaluate the current state of attitude and belief of nursing students toward sexual healthcare and the factors that influence it in Southwest Ethiopia.

**Methods:**

An online cross-sectional survey was conducted in Mizan-Tepi University involving 134 undergraduate nursing students from February 1 to March 10, 2022. The Sexual Attitude and Beliefs Scale (SABS) were used to assess the level of attitudes and beliefs regarding sexual care. Statistical Package for Social Science (SPSS) Version 26 was employed for data analysis. Multivariable linear regression analyses was conducted to identify predictors of attitudes and beliefs regarding sexual healthcare. The significance level was set at p<0.05.

**Results:**

The mean age of the nursing students participating in this study was 28.47±5.2. In our study, mean SABS score of the nursing students was found as 42.3 ± 2.1. The mean score of the SABS items ranged from 1.68±0.93 to 4.37±1.48. Having receiving sexual health education (p<0.001) was significantly associated with attitude and beliefs regarding sexual healthcare.

**Conclusions:**

Ethiopian nursing students have been found to have a negative attitude and beliefs about sexual health care. Because comprehensive sex education is sorely lacking in nursing schools, this scrutiny prevents student nurses from receiving an adequate education. As there is a lack of appropriate sexual health subject matter, it is critical to standardize sexual health education in nursing programs so that nursing students can learn to provide comprehensive care to clients.

## Introduction

Sexual health defined as: “Physical, emotional, and social well-being that relates to one’s sexuality” [1]. Sexual identity, maturity level, personality traits, religious and cultural affiliations all have an impact on how each client defines their sexuality and sexual health [2]. Because of society’s current aversion to discussing sexuality, most women have been disheartened from vigorously interacting with their own sexual issues or communicating with healthcare providers [3].

It is the nurse’s responsibility to provide sexual health services to patients in order to improve people’s lives [4]. Nurse-led sexuality discussions allow patients to discuss their feelings openly while maintaining a high degree of satisfaction between the nurses and the patients [5]. Nurse practitioner applications include "evaluation of the individuals’ sexual health and disclosure of their sexual concerns," as per the American Nursing Association [6].

Most nursing professionals recognize that they play a key role in sexual health care, and there is widespread agreement on the importance of incorporating sexual health education as an indispensable part of the healthcare [7]. Nonetheless, the preparation and eagerness to deal with it in practice appear to be limited [8]. Student nurses were unprepared to participate in sexual healthcare, and few of those polled thought they were skilled to communicate, guide, or create conversations regarding sexual topics [9].

According to sexuality studies, clients were prepared to tell nursing staff about their sex issues; regrettably, nurse practitioners were ineffective in sexual healthcare because their understanding of sexual care was inadequate and their perceptions of sexual healthcare were incorrect [10,11]. Unresolved sexual issues can have an impact on a person’s wellbeing, social interactions, or even medication compliance [12]. As a result of ignoring sexual healthcare needs and failing to provide effective and efficient services, there is a high rate of premature mortality and morbidity [13].

Recognizing nurses’ beliefs and attitudes toward sexuality issues in patients is critical for tailoring initiatives that could enhance their involvement in affective-sexual teaching, which has been shown to have a significant impact on patients [14]. As a result, it is critical for health practitioners to understand their own sexuality values and principles, as well as how they may impact the delivery of comprehensive healthcare [15].

Given the enormous global demand to enhance and preserve sexual health, faculty members have a specific goal of developing nursing workforce who are truly prepared to work with clients who have sexual health issues [16]. As a result, it is critical that student nurses receive adequate knowledge as part of their curriculum in order to work with patients who have sexual health issues [17].

Despite the benefits of recognizing healthcare providers’ attitudes, there is a scarcity of research in this field, with the majority of studies focused on medical graduates and general practitioners [18–20]. The vast majority of research on nurses’ beliefs and attitudes toward client sexual healthcare has been conducted in Western cultural contexts. Because of differences in culture, Ethiopian nursing student’s attitudes and beliefs about sexual healthcare may differ from those of their Western counterparts. As a result, it’s crucial to know Ethiopian nursing students’ attitudes and beliefs about sexual healthcare. This is the first study in Ethiopia to assess nursing students’ attitudes and beliefs about sexual health care. As a result, the study’s aim was to assess nursing students’ current attitudes and beliefs about sexual health care in Southwest Ethiopia, as well as the factors that influence it.

## Materials and methods

### Study design

A cross-sectional online survey study was conducted at Mizan-Tepi University from February 1 to March 10, 2022.

### Study setting

Mizan-Tepi University, situated in Ethiopia’s southwest, has been one of the nation’s best known higher education institutions. The College of Medicine and Health Sciences, which encompasses the departments of Nursing, Medicine, Public Health, Pharmacy, Medical Laboratory Sciences, Midwifery, and Biomedical Sciences, is one of its three campuses. Nursing department has regular, post basic as well as summer programs.

### Study population

The study included all Mizan-Tepi University baccalaureate nursing students who have been capable and willing to give a response to online survey, and also students with one clinical area attachment experience.

### Sample size determination

The sample size was determined using G power 3.1. For multiple regression analysis, a minimum sample size of 134 participants was necessary (effect size 0.15, significance level 0.05, and power of 0.80). We gathered information from 134 nursing students, allowing for a 5% non-response rate.

### Sampling technique

The respondents were chosen using a non-probability sampling method that combined purposive and snowball sampling.

### Data collection tools

The pretested questionnaire was created on Google Forms after a review of various types of literature. The questionnaire is divided into three sections; socio-demographic attributes, Participants’ status of providing sexual healthcare, and “Sexual Attitude and Beliefs Scale” (SABS). Socio-demographic characteristics includes age, sex, nursing year, program, residence, mother educational status and father educational status. Participant’s status of providing sexual healthcare includes the first word that comes to mind when sexuality is mentioned, with whom sexuality is discussed, status of receiving sexual health education, status of clinical sexual health evaluation and causes of not being able to make clinical evaluation. Those issues explained individual understanding of sexuality and sexual healthcare. “The SABS scale consists of 12 items. Participants make markings on a 6 (six)-point Likert type scale for each item ranging from 1 = strongly agree to 6 = strongly disagree. Each item of the scale is evaluated separately. Six of the 12 items (1, 2, 4, 6, 8, 10, 12 items) are scored inversely to avoid bias that can occur when marking the scale. The total score of the scale ranges from 12 to 72. Both the total scale score and the high item scores indicate that the negative attitudes and beliefs regarding sexual health care have increased. Increase in negative attitudes and beliefs also negatively affects the ability of the nurses to assess the sexual problem of the individual and counsel accordingly” [11].

Data quality was ensured during collection, entry, and analysis. The tool’s validity was verified using face validity. The tool was pretested on 10% (14 nursing students) of the actual sample size in the Aman Health Science College 1 week before the actual data collection period and necessary correction have been made to the tool. To ensure consistency, the researcher used English version questionnaire since all the participants were literate. The results showed that the Cronbach’s alpha of the overall SABS was 0.81, which was reliable.

### Data collection procedures

The nursing department gave us the phone numbers of each class’s representatives, and we explained the research’s goals. Having followed that, we used Telegram to send the link to the representatives of each student group, utilizing their contact information. The delegate of each class then communicated the link to their group’s common Telegram location using the purposive sampling method. Using the snowball sampling, students who saw the link in the common Telegram channel send it to their friends through email, and Facebook.

### Operational definition

#### Sexual attitude and beliefs

“Nursing students Sexual Attitude and Beliefs was measured using 12 items on a 6-point Likert scale with value ranging from 12 to 72. The high item scores indicate that the negative attitudes and beliefs regarding sexual health care have increased” [11].

### Data processing and analysis

The data from Google Forms in CSV format was exported to SPSS version 26.0 for analysis. To summarize the data, descriptive and inferential were used. Initially, bivariate linear regression was used to investigate the relationship between attitudes and beliefs about sexual health care and explanatory variables. The explanatory variables are selected from socio-demographic and individual status of proving sexual healthcare variables. Multiple linear regression candidates were variables with p <0.25 in bivariate linear regression. The statistical significance level was set at P<0.05. The assumptions of multiple linear regression were tested prior to analyzing the results. The Kolmogorov-Smirnov test confirmed the normality assumption. The assumption of collinearity was tested using the variance inflation factor (VIF), which had a VIF of less than 5.

### Ethics approval and consent to participate

Approval was granted by the Mizan-Tepi University College of Medicine and Health Sciences Ethics Committee immediately before inquiry. All departmental chiefs were approached. Identities as well as private labels have been eliminated from the layers and findings to protect privacy. The participants were notified of the nature of the project, the perks of the research undertaking, and their right to leave at any time. All the participants provided written informed consent. All of these strategies were conducted in compliance with the standards and rules of the manuscript.

## Results

### Socio-demographic characteristics

The mean age of the nursing students was 28.47 ± 5.2 (min = 20 and max = 45) years. Of these students, 69.4% were male, 50.0% were 4^th^ year, and 44.8% lived in the district. Of their mothers, 67.2.7% were illiterate and of their fathers, 76.9.7% were literate **(Table 1).**

**Table 1:** Socio-demographic characteristics of nursing students (n = 134).

Variables	Category	Frequency	Percentage
**Age** **M 28.47** **SD ±5.265**	20–25	47	35.1
	26–30	48	35.8
	>30	39	29.1
**Sex**	Male	93	69.4
	Female	41	30.6
**Nursing year**	Second year	26	19.4
	Third year	41	30.6
	Fourth year	67	50.0
**Program**	Regular	67	50.0
	Extension	67	50.0
**Residence**	City	47	35.1
	District	60	44.8
	Village	27	20.1
**Mother educational status**	Illiterate	90	67.2
	Literate	44	32.8
**Father educational status**	Illiterate	31	23.1
	Literate	103	76.9

### Participants’ status of providing sexual healthcare

26.9% of nursing students defined sexuality as expressions of “family continuation/reproduction/marriage.” Only 26.9% of students earned sexual education, while 78.4% overlooked sexual health in clinical settings. Most nursing students avert providing sexual healthcare for two reasons: they are embarrassed to do so (58.2%), and they believe it is not their responsibility (23.9%). Furthermore, 46.3% discussed sexual problems primarily with their mates **(Table 2).**

**Table 2:** Nursing students status of providing sexual healthcare (n = 134).

Variables	Category	Frequency	Percentage
**“The first word that comes to mind when sexuality is mentioned”**	Sexual intercourse/sex/joy/pleasure	35	26.1
	Continuation of the family/reproduction/marriage	36	26.9
	Woman–man	23	17.2
	Love	24	17.9
	Sense of privacy/shame	16	11.9
**“With whom sexuality is discussed”**	Friends	62	46.3
	Family members	28	20.9
	No one	44	32.8
**“Status of receiving sexual health education”**	Yes	36	26.9
	No	98	73.1
**“Status of clinical sexual health evaluation”**	No	105	78.4
	Sometimes	16	11.9
	Usually	5	3.7
	Always	8	6.0
**“Causes of not being able to make clinical evaluation on sexual healthcare”**	Feeling shy about providing sexual healthcare	78	58.2
	Thinking that the patient will not take it serious	8	6.0
	The patient is old	8	6.0
	Patient is single	8	6.0
	Thinking that it is not my responsibility	32	23.9

### The level of attitude and belief regarding sexual healthcare

The total SABS score was 42.3 ± 2.1 and the mean scores of the items ranged from 1.68 ± 0.93 to 4.37 ± 1.48. In practice, a high SABS score demonstrated negative attitudes and beliefs towards to the clients’ sexuality issues. The major obstacles to sexual health care were “making time to discuss sexual concerns” (item 6, mean 4.19), being **“**comfortable talking about sexual issues” (item 4, mean 4.37) and “level of understanding how patients’ diseases and treatments might affect their sexuality” (item 2, mean 4.27). One of the least significant barriers was assuming that “discussing sexuality is influential in patients’ healthcare outcomes” (item 1, mean 1.68), being “uncomfortable talking about sexual issues” (item 3, mean 2.50) and “when patients ask sex‐related question, they advise them to discuss it with their physician” (item 7, mean 2.66) **(Table 3).**

**Table 3:** Attitudes and beliefs of nursing students regarding sexual healthcare (n = 134).

Variable	Possible scores	Mean (standard deviation)	Minimum	Maximum
“Discussing sexuality is essential to patients’ health outcomes”	1–6	1.68(SD±0.93)	1.00	5.00
“I understand how my patients’ diseases and treatments might affect their sexuality”	1–6	4.27(SD±1.37)	2.00	6.00
“I am uncomfortable talking about sexual issues”	1–6	2.50(SD±1.39)	1.00	6.00
“I am more comfortable talking about sexual issues with my patients than most of the nurses I work with”	1–6	4.37(SD±1.48)	1.00	6.00
“Most hospitalized patients are too sick to be interested in sexuality”	1–6	3.54(SD±1.34)	1.00	6.00
“I make time to discuss sexual concerns with my patients”	1–6	4.19(SD±1.49)	1.00	6.00
“When patients ask me sex‐related question, I advise them to discuss it with their physician”	1–6	2.66(SD±1.05)	1.00	6.00
“I feel confident in my ability to address patients’ sexual concerns”	1–6	4.10(SD±1.53)	1.00	6.00
“Sexuality is too private an issue to discuss with patients”	1–6	4.04(SD±1.50)	1.00	6.00
“Giving a patient permission to talk about sexual concerns is a nursing responsibility”	1–6	3.28(SD±1.35)	1.00	6.00
“Sexuality should be discussed only if initiated by the patient”	1–6	3.82(SD±1.23)	1.00	6.00
“Patients expect nurses to ask about their sexual concerns”	1–6	3.80(SD±1.50)	1.00	6.00
**Total SABS scale score**	12–72	42.3 (SD±2.1)	40.00	45.00

### Factors associated with attitude and belief regarding sexual healthcare

Multivariable linear regression analyses revealed factors associated with sexual attitude and beliefs among nursing students. In a bivariate linear regression, father educational status, with whom sexuality is discussed, and status of receiving sexual health education were found to be substantially associated with attitude and beliefs regarding sexual healthcare among nursing students at p<0.25. To investigate factors related to attitude and beliefs regarding sexual healthcare, independent variables with p<0.25 in the bivariate linear regression analysis were added to the multivariable linear regression analysis. At a significance level of 0.05, the backward elimination approach was used to choose the variables for the final model.

The findings revealed that status of receiving sexual health education was significantly associated with attitude and beliefs regarding sexual healthcare among nursing students. Accordingly, having receiving education in sexual health decreased negative attitude and beliefs regarding sexual healthcare by -1.949 times compared to those who didn’t receive any education in sexual healthcare (β = -1.949, p <0.001) **(Table 4).**

**Table 4:** Multivariable linear regression analysis regarding sexual healthcare among nursing students (n = 134).

Predictor variable	Unstandardized coefficient		p-value	95% CI	
	Β	SE		Upper	Lower
**Father educational status** Illiterate Literate(reference)					
	-0.074	0.431	0.864	-0.926	0.778
**With whom sexuality is discussed** Friends(reference) Family members No one	0.786 0.650	0.432 0.406	0.071 0.112	-0.068 -0.154	1.640 1.455
**Status of receiving sexual health education** Yes No(reference)					
	-1.949	0.398	0.000**	-2.736	-1.161
				

**p < 0.001.

## Discussion

The total mean SABS score was 42.3.08 ± 2.1, which indicated negative attitudes and beliefs toward sexual healthcare. Negative attitudes and beliefs can impair student nurses’ capability to analyze and consult with people who have sexuality concerns. The SABS total score of the nursing students in this study was similar with a study conducted in Turkey (42.29±3.65) [21].

The finding was higher than studies conducted in Turkey (38.03 ± 8.3) [22], Indonesia (38.04 ± 6.19) [23], Canada (37.48 ± 8.19) [24] and Sweden (40.7 ± 7.8) [25]. These findings suggest that, in comparison to developed nations, our country still has social taboos surrounding sexuality. As a result, this evidence explains why our country’s nursing students are unable to properly analyze their patients’ sexual health, implying that it is a barrier to care provision. In contrast, the current finding was lower than Chinese nurses (45.83 ± 8.14) [26]. Confucius’ teachings have had a significant impact on Chinese thought and culture [27]. According to Confucian philosophy, sexuality is perceived as a solely reproductive role, and discussing sexual problems outside of the married couple is extremely insulting [28]. As a result, conservative attitudes against sexuality concerns are able to impact Chinese nurses’ discipline [26].

First most significant barrier for nursing students when offering sexual healthcare was “feeling more comfortable discussing the patients’ sexual issues with their colleagues.’ The finding was similar to a study conducted in Turkey [22]. According to the current study, nursing students were generally uneasy and reluctant to provide sexual healthcare due to feelings of shyness about providing sexual healthcare and the belief that it was not their responsibility. As a result, student nurses must obtain sexual health education in order to become more cognizant of their own attitudes and beliefs regarding sexuality issues.

Numerous factors determine nurses’ and nursing students’ attitude and belief of sexual healthcare. This study found that those nursing students who didn’t receive sexual healthcare education had more negative attitudes and beliefs regarding sexual healthcare. In other words, it was found that nursing students who received sexual healthcare education faced very few challenges while providing sexual care counselling services to their clients. This finding is similar with studies conducted in Turkey [22,29], Finland [30], Sweden [25], and China [31].

The findings of this study demonstrated no significant relation between sex, and nursing year with attitude and belief regarding sexual healthcare. In contrast, studies revealed that being female [15,21,22] and being first year student [22] had negative attitude and belief toward sexual healthcare.

The current study has some limitations. The study relied on student nurses’ self-reported data, which could lead to social desirability bias. Selection bias as a result of sampling techniques used necessitates a careful interpretation of the results. Furthermore, the investigation only included student nurses from a single organization, which may limit the study’s generalizability to student nurses throughout Ethiopia as well as students from other fields of study at the same institution. Because the study was cross-sectional, it was impossible to determine causation.

## Conclusions

Ethiopian nursing students have been found to have a negative attitude and beliefs about sexual health care. Because comprehensive sex education is sorely lacking in nursing schools, this scrutiny prevents student nurses from receiving an adequate education. As there is a lack of appropriate sexual health subject matter, it is critical to standardize sexual health education in nursing programs so that nursing students can learn to provide comprehensive care to clients. Quite consistent and systematic sexual education in nursing programs is desperately required such that new graduates can confront sexual issues in professional manner and conveniently that reflect their clients’ high standards and trust factor. To ascertain cause-and-effect connections, an experimental study with such a larger sample is needed.

## Supporting information

S1 File Data collection tool. (DOCX)

S2 File SPSS statistics data. (SAV)

## References

[1] World Health Organization. Sexual health and its linkages to reproductive health: an operational approach. 2017. Retrieved from https://www.who.int/reproductivehealth/publications/sexual_health/sh-linkages-rh/en/.

[2] Southard NZ, Keller J. The importance of assessing sexuality: a patient perspective. Clinical journal of oncology nursing. 2009 Apr 1;13(2). doi: 10.1188/09.CJON.213-217 19349268

[3] Bolajoko O.O. "Nurses’ knowledge and Attitudes towards Sexual Health in Al-Gharbyia Governorate, Egypt". IOSR Journal of Nursing and Health Science (IOSR-JNHS), vol. 8, no.06, 2019, pp. 11–21.

[4] Sung SC, Lin YC. Effectiveness of the sexual healthcare education in nursing students’ knowledge, attitude, and self-efficacy on sexual healthcare. Nurse education today. 2013 May 1;33(5):498–503. doi: 10.1016/j.nedt.2012.06.019 22789872

[5] Aaberg V. The state of sexuality education in baccalaureate nursing programs. Nurse education today. 2016 Sep 1;44:14–9. doi: 10.1016/j.nedt.2016.05.009 27429324

[6] Alligood MR. Nursing theorist and their work, eight edition. Missouri: Elsevier. 2014.

[7] Sharon D, Gonen A, Linetsky I. Factors influencing nursing students’ intention to practice sexuality education in their professional work. American Journal of Sexuality Education. 2020 Apr 2;15(2):262–78.

[8] Blakey EP, Aveyard H. Student nurses’ competence in sexual health care: A literature review. Journal of clinical nursing. 2017 Dec;26(23–24):3906–16. doi: 10.1111/jocn.13810 28328169

[9] Kong SK, Wu LH, Loke AY. Nursing students’ knowledge, attitude and readiness to work for clients with sexual health concerns. Journal of Clinical Nursing. 2009 Aug;18(16):2372–82. doi: 10.1111/j.1365-2702.2008.02756.x 19583667

[10] Wang P, Ai J, Davidson PM, Slater T, Du R, Chen C. Nurses’ attitudes, beliefs and practices on sexuality for cardiovascular care: A cross‐sectional study. Journal of clinical nursing. 2019 Mar;28(5–6):980–6. doi: 10.1111/jocn.14692 30338867

[11] Reynolds KE, Magnan MA. Nursing attitudes and beliefs toward human sexuality: collaborative research promoting evidence-based practice. Clinical Nurse Specialist. 2005 Sep 1;19(5):255–9. doi: 10.1097/00002800-200509000-00009 16179857

[12] Southard NZ, Keller J. The importance of assessing sexuality: a patient perspective. Clinical journal of oncology nursing. 2009 Apr 1;13(2). doi: 10.1188/09.CJON.213-217 19349268

[13] Haboubi NH, Lincoln N. Views of health professionals on discussing sexual issues with patients. Disability and rehabilitation. 2003 Jan 1;25(6):291–6. doi: 10.1080/0963828021000031188 12623620

[14] Denno DM, Hoopes AJ, Chandra-Mouli V. Effective strategies to provide adolescent sexual and reproductive health services and to increase demand and community support. Journal of adolescent health. 2015 Jan 1;56(1):S22–41. 10.1016/j.jadohealth.2014.09.012 25528977

[15] Papaharitou S, Nakopoulou E, Moraitou M, Tsimtsiou Z, Konstantinidou E, Hatzichristou D. Exploring sexual attitudes of students in health professions. The Journal of Sexual Medicine. 2008 Jun;5(6):1308–16. doi: 10.1111/j.1743-6109.2008.00826.x 18410302

[16] Jolley S. Promoting teenage sexual health: an investigation into the knowledge, activities and perceptions of gynaecology nurses. Journal of advanced nursing. 2001 Oct 16;36(2):246–55. doi: 10.1046/j.1365-2648.2001.01965.x 11580799

[17] Thistle S, Ray C. Sex and relationships education: The role of the school nurse. Nursing Standard (through 2013). 2002 Sep 18;17(1):44. doi: 10.7748/ns2002.09.17.1.44.c3269 12360740

[18] Merrill JM, Laux LF, Thornby JI. Why doctors have difficulty with sex histories. Southern medical journal. 1990 Jun 1;83(6):613–7. doi: 10.1097/00007611-199006000-00004 2356491

[19] Gott M, Hinchliff S, Galena E. General practitioner attitudes to discussing sexual health issues with older people. Social science & medicine. 2004 Jun 1;58(11):2093–103. doi: 10.1016/j.socscimed.2003.08.025 15047069

[20] Shindel AW, Parish SJ. CME information: Sexuality education in North American medical schools: Current status and future directions (CME). The Journal of Sexual Medicine. 2013 Jan 1;10(1):3–18. doi: 10.1111/j.1743-6109.2012.02987.x 23343168

[21] Güven ŞD, Çelik GK. Evaluation of nursing students’ attitudes and beliefs regarding sexual care. Androl Bul 2021;23:7–12. 10.24898/tandro.2021.02703.

[22] Güdül Öz H, Balcı Yangın H, Ak Sözer G. Attitudes and beliefs of nursing students toward sexual healthcare: a descriptive study. Perspect Psychiatr Care. 2021; 1–7. 10.1111/ppc.12780. 33811646

[23] Attitudes Afiyanti Y., belief, and barriers of Indonesian oncology nurses on providing assistance to overcome sexuality problem. Nurse Media Journal of Nursing. 2017 Dec 1;7(1):15–23.

[24] Magnan MA, Reynolds K. Barriers to addressing patient sexuality concerns across five areas of specialization. Clinical Nurse Specialist. 2006 Nov 1;20(6):285–92. doi: 10.1097/00002800-200611000-00009 17149019

[25] Saunamäki N, Andersson M, Engström M. Discussing sexuality with patients: nurses’ attitudes and beliefs. Journal of advanced nursing. 2010 Jun;66(6):1308–16. doi: 10.1111/j.1365-2648.2010.05260.x 20384642

[26] Zeng YC, Li Q, Wang N, Ching SS, Loke AY. Chinese nurses’ attitudes and beliefs toward sexuality care in cancer patients. Cancer Nursing. 2011 Mar 1;34(2):E14–20. doi: 10.1097/NCC.0b013e3181f04b02 20921885

[27] Woo JS, Brotto LA, Gorzalka BB. The role of sexuality in cervical cancer screening among Chinese women. Health Psychology. 2009 Sep;28(5):598. doi: 10.1037/a0015986 19751086

[28] Khoo SB. Impact of cancer on psychosexuality: cultural perspectives of Asian women. International journal of nursing practice. 2009 Dec;15(6):481–8. doi: 10.1111/j.1440-172X.2009.01797.x 19958401

[29] ALCAN AO, ÇETİN S, AK ES, ÇULHA Y, ÖZBAŞ A. Determination of Nurses’ Attitudes and Beliefs on Sexual Care Towards Urology Patients. Yeni Üroloji Dergisi.;16(1):60–7.

[30] Hautamäki K, Miettinen M, Kellokumpu-Lehtinen PL, Aalto P, Lehto J. Opening communication with cancer patients about sexuality-related issues. Cancer nursing. 2007 Sep 1;30(5):399–404. doi: 10.1097/01.NCC.0000290808.84076.97 17876186

[31] Zeng YC, Liu X, Loke AY. Addressing sexuality issues of women with gynaecological cancer: Chinese nurses’ attitudes and practice. Journal of Advanced Nursing. 2012 Feb;68(2):280–92. doi: 10.1111/j.1365-2648.2011.05732.x 21658098

