# Supplementary material for: Nursing students’ attitudes and beliefs regarding sexual healthcare in Ethiopia: An online cross-sectional study
Source: PLoS One. 2022 Dec 7;17(12):e0278760. doi: 10.1371/journal.pone.0278760 (PMC9728890; doi:10.1371/journal.pone.0278760)
Supplement: S1 File — (DOCX) [file pone.0278760.s001.docx]

**Attitudes and beliefs of nursing students toward sexual healthcare in southwest Ethiopia: An online cross-sectional study**

**Socio-demographic characteristics**

1. Age
2. Sex 1. Male 2. Female
3. Nursing year 1. 2nd year 2. 3^rd^ year 3. 4^th^ year
4. Program 1. Regular 2. Extension
5. Residence for a long time 1. City 2. District 3. Village
6. Mother educational status 1. Can’t read and write 2. Read and write 3. Primary 4. Secondary 5. College and above
7. Father educational status 1. Can’t read and write 2. Read and write 3. Primary 4. Secondary 5. College and above

**Participants' status of providing sexual healthcare (MCQ)**

1. The first word that comes to mind when sexuality is mentioned 1. Sexual intercourse/sex/joy/pleasure 2. Continuation of the family/reproduction/marriage 3. Woman–man 4. Love 5. Sense of privacy/shame
2. With whom sexuality is discussed 1. Friends 2. Family members 3. No one
3. Status of receiving sexual health education 1. Yes 2. No
4. Status of clinical sexual health evaluation 1. No 2. Sometimes 3. Usually 4. Always
5. Causes of not being able to make clinical evaluation on sexual healthcare 1.Thinking that my sexual health knowledge is inadequate 2. Feeling shy about providing sexual healthcare 3. Thinking that the patient will not take it serious 4. The patient is old 5. The patient is of the opposite sex 6. Patient is single 7. Thinking that it is not my responsibility

**Attitudes and beliefs of nursing students regarding sexual healthcare**

**1=strongly agree to 6. Strongly diagree**

1. Discussing sexuality is essential to patients' health outcomes

2. I understand how my patients' diseases and treatments might affect their sexuality

3. I am uncomfortable talking about sexual issues

4. I am more comfortable talking about sexual issues with my patients than most of the nurses I work with

5. Most hospitalized patients are too sick to be interested in sexuality

6. I make time to discuss sexual concerns with my patients

7. When patients ask me sex‐related question, I advise them to discuss it with their physician

8. I feel confident in my ability to address patients' sexual concerns

9. Sexuality is too private an issue to discuss with patients

10. Giving a patient permission to talk about sexual concerns is a nursing responsibility

11. Sexuality should be discussed only if initiated by the patient

12. Patients expect nurses to ask about their sexual concerns
